# Interplay between diffusion and magnon-drag thermopower in pure iron and dilute iron alloy nanowire networks

**DOI:** 10.1038/s41598-023-36391-y

**Published:** 2023-06-07

**Authors:** Nicolas Marchal, Tristan da Câmara Santa Clara Gomes, Flavio Abreu Araujo, Luc Piraux

**Affiliations:** Institute of Condensed Matter and Nanosciences, Université catholique de Louvain, Place Croix du Sud 1, 1348 Louvain-la-Neuve, Belgium

**Keywords:** Nanowires, Condensed-matter physics, Thermoelectric devices and materials

## Abstract

Results of measurements on the thermoelectric power of 45 nm diameter interconnected nanowire networks consisting of pure Fe, dilute FeCu and FeCr alloys and Fe/Cu multilayers are presented. The thermopower values of Fe nanowires are very close to those found in bulk materials, at all temperatures studied between 70 and 320 K. For pure Fe, the diffusion thermopower at room temperature, estimated to be around − 15 $\upmu$V/K from our data, is largely supplanted by the estimated positive magnon-drag contribution, close to 30 $\upmu$V/K. In dilute FeCu and FeCr alloys, the magnon-drag thermopower is found to decrease with increasing impurity concentration to about 10 $\upmu$V/K at 10$\%$ impurity content. While the diffusion thermopower is almost unchanged in FeCu nanowire networks compared to pure Fe, it is strongly reduced in FeCr nanowires due to pronounced changes in the density of states of the majority spin electrons. Measurements performed on Fe(7 nm)/Cu(10 nm) multilayer nanowires indicate a dominant contribution of charge carrier diffusion to the thermopower, as previously found in other magnetic multilayers, and a cancellation of the magnon-drag effect. The magneto-resistance and magneto-Seebeck effects measured on Fe/Cu multilayer nanowires allow the estimation of the spin-dependent Seebeck coefficient in Fe, which is about − 7.6 $\upmu$V/K at ambient temperature.

## Introduction

In ferromagnetic metals, electrons are scattered by spin waves. When these materials are subjected to a temperature gradient, a magnon current flows from the hot region to the cold region, interacting with the electronic system. Similar to the scattering by phonons which leads to phonon drag effects, the electron-magnon interaction can produce magnon-drag effects that contributes positively to the Seebeck coefficient. The absolute thermoelectric power of a magnetic material is approximately given by the sum of three independent contribution:

1 $$\begin{aligned} S = S_\text {d} + S_\text {p} + S_\text {md} , \end{aligned}$$

where $S_\text {d}$ is the conventional electron-diffusion part, $S_\text {p}$ is the phonon-drag contribution, and $S_\text {md}$ is the magnon-drag contribution. The diffusion thermopower in a metal arises from the nonequilibrium of the Fermi-Dirac distribution of the electrons caused by a thermal gradient. According to the Mott formula^1^ one can write:

2 $$\begin{aligned} S_\text {d} = -\frac{\pi ^2}{3} \frac{k_\text {B}^2 T}{e} \left[ \frac{\delta \text {ln}\lambda (\varepsilon )}{\delta \varepsilon } + \frac{\delta \text {ln}\Sigma (\varepsilon )}{\delta \varepsilon } \right] _{\varepsilon _\text {F}} \end{aligned}$$

where *e* is the elementary electronic charge, $\lambda (\varepsilon )$ is the mean free path of electrons on a Fermi surface of area $\Sigma$, and the derivatives are evaluated at the Fermi energy. The diffusion thermopower is thus very sensitive both to changes in the electronic structure and to the mechanisms which scatter the electrons. From previous works, it was found that the theory of magnon-drag follows closely that of phonon-drag^1^ and that $S_\text {md}$ can be expressed as^1–3^

3 $$\begin{aligned} S_\text {md} = \frac{2}{3} \frac{C_\text {m}}{ne} \frac{1}{1+\tau _\text {em} / \tau _\text {m}}\text {,} \end{aligned}$$

where $\tau _\text {em}$ is the scattering time for magnon-electron collisions, $\tau _\text {m}$ the total momentum relaxation time for magnons, *n* the electronic density, and $C_\text {m}$ the magnon specific heat capacity per unit volume. Despite the experimental and theoretical work carried out over the past decades on different materials, it is still difficult to obtain experimental evidence for the existence of magnon-drag effects. One of the reasons is that the separation of thermoelectric power into its different components is relatively complex. In pioneering work, Blatt et al.^4^ measured the thermopower in iron over a wide temperature range and concluded that in Fe, magnon-drag plays a dominant role. Although it is expected that magnon-drag is progressively reduced by external magnetic field, few experimental results have been obtained, showing effects of relatively small amplitudes^2,5^. Subsequent studies on thin film and bulk iron and Fe-based alloys have highlighted the significant contribution of magnon-drag to thermopower^3,6,7^. Besides, evidence for magnon-drag effect in NiFe wires was provided by measurements made on thermopile-like device^8^. It has also been proposed a spin transfer mechanism for magnon-drag thermopower in bulk conducting ferromagnets^9^. More recently, a large magnon-drag contribution to the thermopower has been reported in antiferromagnetic Li-doped MnTe^10^. Also, magnon-drag thermoelectric effect in ferromagnets with a skyrmion structure has been studied theoretically^11^. Furthermore, the emergence of spin-caloritronics and new effects associated with the coupling between charge, spin and heat currents has created a new interest in the study of thermoelectricity in ferromagnetic heterostructures. Among these, the spin Seebeck effect resulting from the interaction between the thermally induced magnonic spin current in the ferromagnet and the generation of a (inverse) spin Hall voltage in an adjacent normal metal has received particular attention^12–14^. On the other hand, ferromagnetic nanowires obtained by electrochemical deposition using nanoporous templates have received a lot of attention in the last decades because this fabrication approach is very versatile, allowing the study of different magnetic nanowire systems, such as single nanowires, parallel nanowire arrays and interconnected nanowire networks^15–21^. In addition, this synthesis approach allows easy fabrication of magnetic alloys of controlled composition as well as multilayer systems where the current flows perpendicular to the plane of the layers (CPP configuration), which is a suitable geometry to investigate giant magneto-transport properties^16,22–24^. Interconnected nanowire networks are particularly suitable for thermopower measurements. Indeed, in this system, electrical and thermal currents flow globally in the plane of the crossed nanowire film following zigzag paths along the nanowire axes^25,26^. This configuration greatly reduces thermal contact resistance issues, a major source of error when the thermal gradient is established in the out-of-plane direction of nanoporous membranes containing parallel nanowire arrays, due to the thinness of the porous templates. The giant magneto-Seebeck effects recently reported in magnetic multilayers made from nanowire networks have made it possible to extract fundamental spin-caloritronic parameters such as the spin-dependent Seebeck coefficients and to realize magnetically activated thermoelectric switches^25,27,28^.

**Figure 1:**
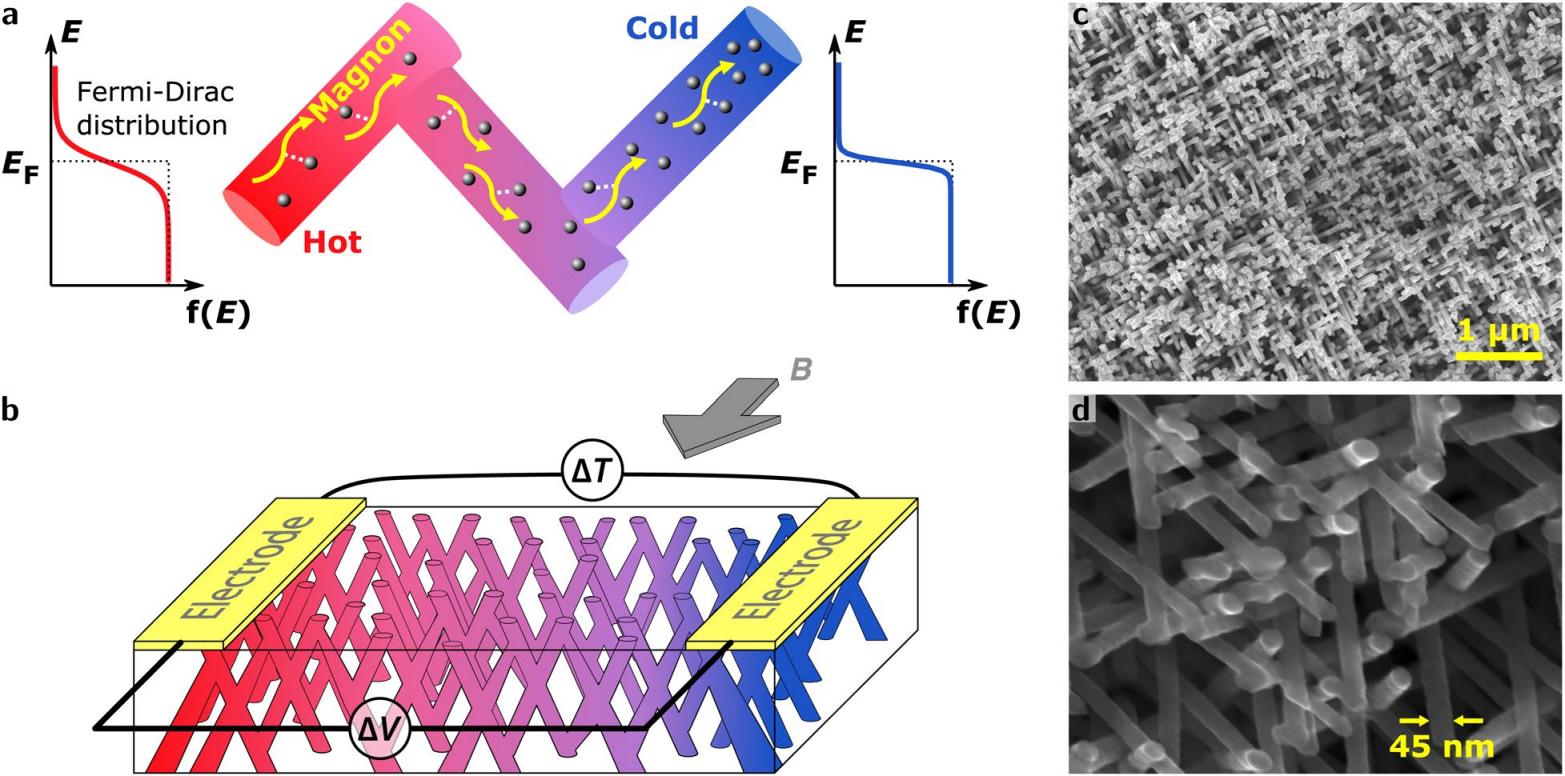
Thermopower of interconnected Fe-based NW networks. (**a**) Difference in the Fermi-Dirac distribution width at the hot and cold extremes of the sample causing the diffusion thermopower and schematics of the magnon-drag effect in interconnected magnetic nanowires. (**b**) Device configuration for measurement of the Seebeck coefficient in interconnected NW network film; the magnetic field ***B*** is along the in-plane direction of the CNW film. (**c**,**d**) SEM images at two magnifications of the self-supported interconnected Fe NWs showing the top view of the CNW network with a 45 nm diameter and a 20$\%$ packing density.

Here, we determine the respective contributions of magnon-drag and diffusion thermopower in 45 nm diameter interconnected nanowire networks (see Fig. 1a) made of pure Fe, dilute FeCu and FeCr alloys and Fe/Cu multilayers. The results of measurements carried out as a function of temperature, magnetic field and impurity concentration are compared with those previously obtained on bulk materials. The analysis highlights the influences of the nature and concentration of impurities and nanostructuring on the contributions of charge carrier diffusion and magnon-drag to thermopower in iron and its alloys.

## Materials and methods

The polycarbonate (PC) porous membranes with interconnected pores have been fabricated by exposing a 22-$\upmu$m-thick PC film to a two-step irradiation process^29,30^. The topology of the membranes was defined by exposing the film to a first irradiation step at two fixed angles of − 25$^\circ$ and $+$25$^\circ$ with respect to the normal axis of the film plane. After rotating the PC film, in the plane by 90$^\circ$, the second irradiation step took place at the same fixed angular irradiation flux to finally form a three-dimensional (3D) nanochannel network. Then, the latent tracks were chemically etched following a previously reported protocol^31^ to obtain 3D porous membranes with pores of 45 nm diameter and volumetric porosity of $20\%$. Next, the PC templates were coated on one side using an e-beam evaporator with a metallic Cr(3 nm)/Au(400 nm) bilayer to serve as cathode during the electrochemical deposition. Each crossed nanowire (CNW) network partially fills the 3D porous PC membrane, typically around $50\%$ of the total pore volume. Pure Fe NW networks were synthesized at room temperature in the potentiostatic mode using a Ag/AgCl reference electrode and a Pt counter electrode from an electrolyte solution composed of 0.5 M FeSO$_4$·7 H$_2$O $+$ 0.485 M H$_3$BO$_3$. The pH acidity of the Fe-based solution was set to 2 and a deposition potential of − 1.2 V was used. Dilute FeCu alloy NW networks with a Cu content $\le$ 10$\%$ were grown by adding between 5 and 55 mM of CuSO$_4$·5H$_2$O into an electrolyte solution containing 0.5 M FeSO$_4$·7 H$_2$O $+$ 0.485 M H$_3$BO$_3$. Interconnected FeCr alloy NWs (Cr content $\le$ 10 at. $\%$) were obtained by adding between 5 and 50 mM of CrCl$_3$·4 H$_2$O into an electrolyte solution containing 0.5 M of FeSO$_4$·7 H$_2$O and 0.485 M H$_3$BO$_3$. The deposition potential was $-1.2$ V for both FeCu and FeCr NW networks. In addition, Fe/Cu multilayer (ML) nanowires were made from a single-sulfate bath using a pulsed electrodeposition technique in the potentiostatic mode as previously described^25,32^. The electrolyte composition was 1.2 M FeSO$_4$·7 H$_2$O $+$ 6 mM CuSO$_4$·5 H$_2$O $+$ 0.485 M H$_3$BO$_3$ $+$ 0.45 M (NH$_4$)$_2$SO4. The pH was around 3 (unadjusted). The Fe rich and Cu layers were deposited at − 1.2 V and − 0.4 V vs. Ag/AgCl reference electrode, respectively. Using these experimental conditions for electrochemical deposition and following a procedure described elsewhere^16^, the deposition rates of each metal were previously determined from the pore filling time. According to this calibration, the deposition time was adjusted to 200 ms and 25 s for the Fe and Cu layers, leading approximately to a Fe(7 nm)/Cu(10 nm) multilayer stack made of 400 bilayers. The Fe/Cu multilayer only partially fills the porous membrane. Thermopower and magnetoresistance measurements of the ferromagnetic CNWs networks were performed as a function of temperature using home-made set-ups, as described elsewhere^25,27^. For conducting electrical and thermoelectric transport measurements, the cathode was locally removed by plasma etching to create a two-probe configuration (Fig. 1b). The electrical contacts are made by Ag paint on the metallic electrodes. Typical dimensions of the CNW film samples are 10 mm long, 2 mm width and 0.022 mm thick. In this system, the current is injected in the macroscopic direction of the film plane through the network of NWs thanks to the high degree of electrical connectivity of the CNWs. The typical resistance values of the prepared specimens are in the range of few tens of ohms. For each sample, the input power is kept below 0.1 $\upmu$W to avoid self-heating, and the resistance was measured within its ohmic resistance range with a resolution of one part in 10$^{5}$. As described in previous works^25,27^, the Seebeck coefficient was measured by attaching one end of the sample to the copper sample holder using silver paint and a resistive heater to the other end. The voltage leads were made of thin Chromel P wires, and the contribution of the leads to the measured thermoelectric power was subtracted out using the recommended values for the absolute thermopower of Chromel P. The temperature gradient was monitored with a small-diameter type E differential thermocouple. A typical temperature difference of 1 K was used in the measurements. Electrical and thermoelectric measurements were performed under vacuum. For Fe/Cu ML NWs, the magnetic variation of the resistance and Seebeck coefficient is measured by sweeping an external magnetic field between − 8 and 8 kOe along the in-plane direction of the CNW films. On the other hand, for FeCu and FeCr NWs, thermopower measurements were made at zero magnetic field, as these materials exhibit magneto-Seebeck effects of less than 0.1$\%$ at $H =$ 8 kOe. The temperature of the samples can be varied from 10 to 320 K. The interconnected NW structure was characterized using a field emission scanning electron microscope (FE-SEM) after chemical dissolution of the polymer template. The NW networks form an exact replica of the 3D pristine porous film and were found to be mechanically stable and self-supported, as illustrated by the SEM images shown in Fig. 1c,d. Energy-dispersive X-ray spectroscopy (EDX) has provided the chemical composition of the Fe-based dilute alloy NWs, expressed as atomic percentage in this work. For Fe/Cu multilayers, Cu impurity is incorporated only to a very limited content (less than 5$\%$) in iron layers, as also evaluated by EDX analysis.

## Results and discussions

**Figure 2:**
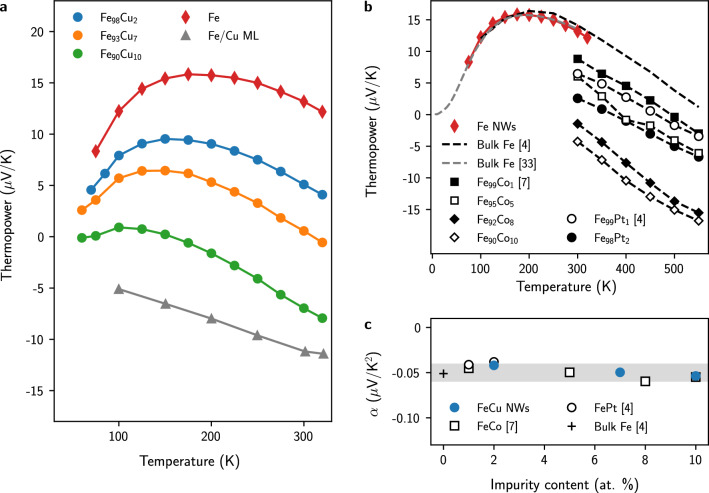
Measured Seebeck coefficient of nanowire networks made of pure iron and iron-based dilute alloys. (**a**) Temperature dependence of the thermopower *S* of 45 nm diameter NW networks made of pure Fe, Fe-rich FeCu alloys and Fe(7 nm)/Cu(10 nm) multilayer. (**b**) Comparison between the *S*(*T*) curves obtained on pure Fe nanowires, bulk Fe and dilute Fe-based alloys. (**c**) Estimated values of the slope $\alpha$ of the linear decay of *S*(*T*) for *T* $\ge$ 200 K for bulk Fe and dilute Fe-based alloys with impurity content less than 10$\%$. The shaded area shows the values of $\alpha$ in the range − 0.05 ± 0.01 $\upmu$V/K$^2$.

Figure 2a shows the temperature evolution between 70 and 320 K of the thermopower of various nanowire networks made of pure Fe, Fe$_{100-x}$Cu$_x$ alloys (with $x =$ 2, 7, 10) and a Fe(7 nm)/Cu(10 nm) multilayer. For pure Fe nanowires, the thermopower values are positive and show a maximum at 16 $\upmu$V/K around $T =$ 200 K. Overall, it appears that the introduction of Cu impurities in Fe leads to a reduction of the total measured thermopower which even becomes negative for Fe$_{90}$Cu$_{10}$ NWs over almost the whole temperature range ($\sim$ − 7 $\upmu$V/K at 300 K). For Fe(7 nm)/Cu(10 nm) multilayer NWs, the measured thermopower is negative and varies almost linearly with temperature. Besides, the experimental results obtained on 45 nm diameter Fe CNWs are very similar to those previously reported for bulk iron^4,33^, as shown in Fig. 2b. This correspondence indicates that the contribution of phonon-drag to the thermopower is negligible in Fe because this component is very much affected by nanostructuring, as recently demonstrated for porous cobalt^3^. It appears therefore that only electron diffusion and magnon-drag contributions of the thermopower should be taken into account to describe the thermoelectric properties in Fe, as previously highlighted in the pioneering work of Blatt^4^. The high-temperature data in Fig. 2b shows a quasi-linear decay of *S* up to about 500 K, with a slope $\alpha$ $\sim$ − 0.05 $\upmu$V/K$^2$. Interestingly, the same linear relationship is observed above *T* $\sim$ 200 K in nanowires formed from dilute FeCu alloys (see Fig. 2a) with a slope similar to that obtained in bulk Fe, as shown in Fig. 2c. This trend is corroborated by previous results obtained in dilute FeCo and FePt bulk alloys with impurity content less than 10$\%$ in the same temperature range^4,7^, also reported in Fig. 2c, showing that the slopes for all these Fe-based alloys correspond approximately to $\alpha$ $\sim$ − 0.05 ± 0.01 $\upmu$V/K$^2$.

**Figure 3:**
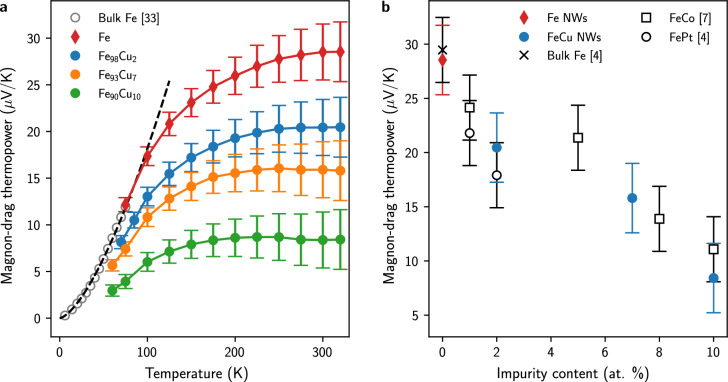
Magnon-drag thermopower of pure iron and iron-based dilute alloy nanowires. (**a**) Temperature dependence of the estimated magnon-drag thermopower $S_\text {md}$ of NW networks made of pure Fe and dilute FeCu alloys and of bulk Fe at low temperature (opened symbols from^33^). All the data were obtained by subtracting the same linear negative contribution $\alpha T$ (with $\alpha = -0.05 \pm 0.01\, \upmu$V/K$^2$) to the measured thermopower. The dashed line is the result of calculation with Eq. (2) leading to $S_\text {md}$ $=$ $\beta T^{3/2}$ with $\beta \sim$ 0.018 $\upmu$V/K$^{5/2}$. (**b**) Evolution of the estimated values of $S_\text {md}$ at room temperature for various dilute Fe-based alloys: FeCu NWs (present work), FeCo^7^ and FePt^4^.

Figure 3a shows the temperature-dependent magnon-drag thermopower $S_\text {md}$ for Fe and Fe$_{100-x}$Cu$_x$ nanowire networks obtained by subtracting the diffusive contribution to the measured thermopower. Overall, these results indicate that the positive magnon-drag thermopower, which reaches the estimated value of 30 $\upmu$V/K at room temperature (RT) in pure iron, is progressively decreased as a result of the increasing impurity content in Fe, while the diffusion thermopower is little affected in these dilute alloys. On the other hand, it is very likely that the contribution of magnon-drag to the thermopower is negligible in Fe/Cu multilayer CNWs (see Fig. 2a), not only because of the presence of a few percent of Cu impurities in the nm-thick ferromagnetic layers but also because of the linear evolution of the measured thermopower with temperature. Due to the current-perpendicular-to-plane (CPP) configuration of the Fe/Cu multilayer, $S_\text {Fe/Cu}$ is mainly determined by the thermopower of the ferromagnetic metal, which is significantly higher than that of Cu, as already demonstrated in previous studies on Co/Cu, CoNi/Cu and NiFe/Cu multilayers^27^. Indeed, the Seebeck coefficient of Fe/Cu multilayer in the perpendicular direction to the layers can be expressed from the corresponding transport properties using Kirchhoff’s rules as^27,34^

4 $$\begin{aligned} S_\text {Fe/Cu} = \frac{S_\text {Cu} \rho _\text {Cu} + \gamma S_\text {Fe} \rho _\text {Fe}}{\rho _\text {Cu} + \gamma \rho _\text {Fe}}\text {.} \end{aligned}$$

Here $S_\text {Fe}$, $S_\text {Cu}$ and $\rho _\text {Fe}$, $\rho _\text {Cu}$ represent the thermopower and the electrical resistivity of Fe and Cu and $\gamma =$ $t_\text {Fe}$/$t_\text {Cu}$ is the thickness ratio of Fe and Cu layers. From Eq. (4), using the experimental value at RT for Fe/Cu multilayer with $\gamma$ = 0.7 (*S* $\sim$ − 11 $\upmu$V/K), and the bulk values for $S_\text {Cu}$, $\rho _\text {Fe}$ and $\rho _\text {Cu}$, we estimate the value of $S_\text {Fe}$ $\sim$ − 14.5 $\upmu$V/K at *T* = 300 K. Besides, the thermopower of iron evaluated in this way depends little on the exact value of $\gamma$. Indeed, the RT values for $S_\text {Fe}$ are − 15.5 $\upmu$V/K and − 13.5 $\upmu$V/K for $\gamma$ = 0.5 and $\gamma$ = 1, respectively. As expected, the diffusion thermopower of Fe deduced from Eq. (4) is more negative than the value measured in the Fe/Cu multilayer since the thermopower of elemental copper is positive (1.8 $\upmu$V/K at *T* = 300 K). It is worth noting that this estimate of $S_\text {Fe}$ coincides remarkably well with that obtained at RT using the average value of $\alpha$ extracted from the data of Fig. 2c. Overall, our analysis shows that the diffusion thermopower is negative in Fe, as it is also in Co and Ni, even though these 2 other metals have higher absolute values, near − 30 $\upmu$V/K and − 20 $\upmu$V/K at *T* = 300 K, respectively^1,3,35,36^.

All $S_\text {md}$(*T*) curves in Fig. 3a show a monotonic increase with temperature followed by a trend towards saturation or a broad peak. Saturation or the maximum in the temperature variation of the magnon-drag thermopower occurs at a lower temperature the higher the Cu content in the alloy. While for pure Fe, saturation occurs around RT, it appears as early as 150 K for the Fe$_{90}$Cu$_{10}$ sample. The opened symbols in Fig. 2a correspond to the low-temperature evolution of the magnon-drag thermopower in bulk Fe, as obtained by subtracting the same negative linear component $-\alpha T$ from the experimental data in^33^. In Fig. 3a, it is also shown that the data follow the predicted $T^{3/2}$ law for the magnon-drag thermopower which, according to Eq. (3), can be expressed as^2,3^

5 $$\begin{aligned} S_\text {md} = \frac{2}{3ne} \frac{k_\text {B}^{5/2}}{4\pi ^2} \frac{T^{3/2}}{D^{3/2}} L(0) \frac{1}{1+\tau _\text {em} / \tau _\text {m}}\text {,} \end{aligned}$$

with $k_\text {B}$ the Boltzmann constant, *D* the spin wave stiffness and $L(0) =$ 4.45. Assuming that magnons are scattered dominantly by electrons, i.e. the factor (1 $+$ $\tau _\text {em} / \tau _\text {m}$) is close to 1^3,7^, the dashed line in Fig. 3a represents the calculation using Eq. (5) with $D =$ 245 meV $\mathring{A}^2$, in agreement with the values reported in the literature^37,38^. Equation (5) thus predicts an increase in the form $\beta T^{3/2}$ with increasing temperature with $\beta \sim$ 0.018 $\upmu$V/K$^{5/2}$, which is also close to the value of $\beta \sim$ 0.016 $\upmu$V/K$^{5/2}$ previously reported by Blatt^4^. In addition, it should be noted that the magnon-drag thermopower of the 45 nm diameter Fe nanowires above $T =$ 75 K are perfectly in line with the data obtained on bulk Fe in the low temperature range. Figure 3b shows the evolution of the magnon-drag thermopower $S_\text {md}$ at RT as a function of impurity content in FeCu, FeCo and FePt alloys. Again, the data in this figure were obtained by subtracting the experimental values from a single value of − 15 $\upmu$V/K corresponding to the common diffusion thermopower for all these dilute iron-based alloys evaluated at $T =$ 300 K. Although the values differ somewhat between the different types of alloys, the same trend can be observed. The magnon-drag thermopower is progressively reduced as the impurity content increases, from a value close to 30 $\upmu$V/K in pure Fe to values near 10 $\upmu$V/K for Fe$_{90}$Cu$_{10}$ and Fe$_{90}$Co$_{10}$ alloys. Despite the limited theoretical work describing the scattering of spin waves by the defects^39,40^, it is likely that the reduction of magnon-drag thermopower in both bulk and nanowire iron can be attributed to an increase in magnon-impurity scattering.

**Figure 4:**
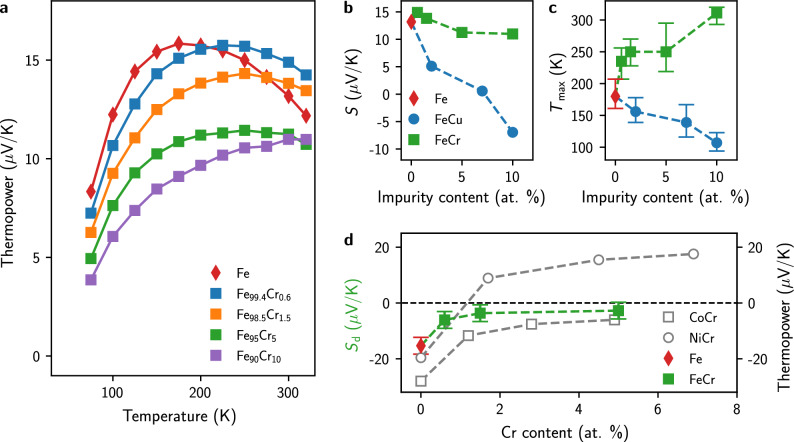
Thermoelectric characteristics of diluted FeCr alloy nanowires. (**a**) Temperature dependence of the thermopower *S* of dilute FeCr alloy NWs compared to pure Fe NWs. (**b**) Variation of the RT Seebeck coefficient of FeCr and FeCu NWs as a function of impurity content. (**c**) Evolution of the maxima in the *S*(*T*) curves for FeCu and FeCr alloys vs impurity content. (**d**) Comparison between the variation of the estimated diffusion thermopower $S_\text {d}$ of FeCr NWs as a function of Cr content and the total measured Seebeck coefficient of NiCr and CoCr NWs from^41^. All the data in (**d**) are at room temperature.

Figure 4 shows the temperature evolution between 70 and 320 K of the thermopower of FeCr NWs for Cr concentration up to 10$\%$. The results obtained for these dilute FeCr alloys are profoundly different from those discussed previously on Figs. 2 and 3. On the one hand, the reduction of *S* following the introduction of Cr impurities is significantly lower than for the other impurity elements and the thermopower remains positive at all temperatures even for the Fe$_{90}$Cr$_{10}$ sample. Moreover, for low Cr concentrations ($x =$ 0.6 and 1.5), there is even a significant increase in *S* compared to pure Fe in the high-temperature range, e.g. *S* rises from $\sim$ 12 $\upmu$V/K in pure Fe at $T =$ 320 K to $\sim$ 14 $\upmu$V/K in Fe$_{99.4}$ Cr$_{0.6}$. The very marked differences with FeCu NWs are also illustrated in Fig. 4b,c. Figure 4b shows the variation of the RT thermopower as a function of impurity concentration for both types of alloys, where a much greater decrease in thermopower can be observed for FeCu alloys compared to FeCr alloys. Figure 3c shows the contrasting shifts of the maxima in the experimental *S*(*T*) curves for FeCu and FeCr alloy NWs. The results of Fig. 4c show that the maximum in the *S*(*T*) curve for pure Fe at $T =$ 180 K drops to $T =$ 100 K for Fe$_{90}$Cu$_{10}$ while for FeCr alloys the maximum increase for increasing Cr content and reaches around RT for Fe$_{90}$Cr$_{10}$.

This contrasting behavior for dilute FeCr alloys originates from a strong modification of the diffusion thermopower, in agreement with previous results obtained on CoCr and NiCr NWs^41^. Indeed, as shown in Fig. 4d, the measured Seebeck coefficient of NiCr at RT suddenly changes sign from negative (− 20 $\upmu$V/K for pure Ni) to relatively large positive values with the addition of Cr impurities in Ni (18 $\upmu$V/K for the Ni$_{93}$Cr$_{7}$). Similarly, for CoCr CNWs the measured thermopower at RT drops rapidly from − 28 $\upmu$V/K for pure Co to much smaller negative values approaching − 5 $\upmu$V/K for Co$_{95}$Cr$_{5}$ CNWs (see Fig. 4d). These results can be explained on the basis of a virtual bound state passing through the Fermi level in the spin up band so that the minority spin dominates the electrical conduction^42–44^. We believe that these results obtained on the NW networks made of dilute FeCr alloys are also consistent with pronounced changes in the density of states for the majority spin electrons. Using a very narrow temperature range between 250 and 320 K, we have attempted to extract for each of the *S*(*T*) curves obtained on FeCr NWs the slope of the thermopower decay and report the evolution of the diffusion thermopower contribution thus estimated at RT as a function of the Cr concentration. Despite the relatively high uncertainty in the estimated values of thermopower diffusion, it appears from Fig. 4d that the overall behavior for dilute FeCr alloy CNWs is in very reasonable agreement with those previously obtained for NiCr and CoCr CNWs^41^. Furthermore, assuming that the diffusion thermopower is negligible for Fe$_{90}$Cr$_{10}$ CNWs, as suggested in Fig. 4d, the *S*(*T*) curve obtained for this alloy (see Fig. 4a) is dominated by the magnon-drag component over the whole temperature range. Besides, the thermopower at RT (around 10 $\upmu$V/K) coincides remarkably well with the magnon-drag contribution estimated for Fe$_{90}$Cu$_{10}$ and Fe$_{90}$Co$_{10}$ alloys which are shown in Fig. 3b. The results obtained on the FeCr CNWs reinforce the overall consistency of our analysis on the respective contributions of electron diffusion and magnon-drag to thermopower in dilute iron alloys and pure iron.

**Figure 5:**
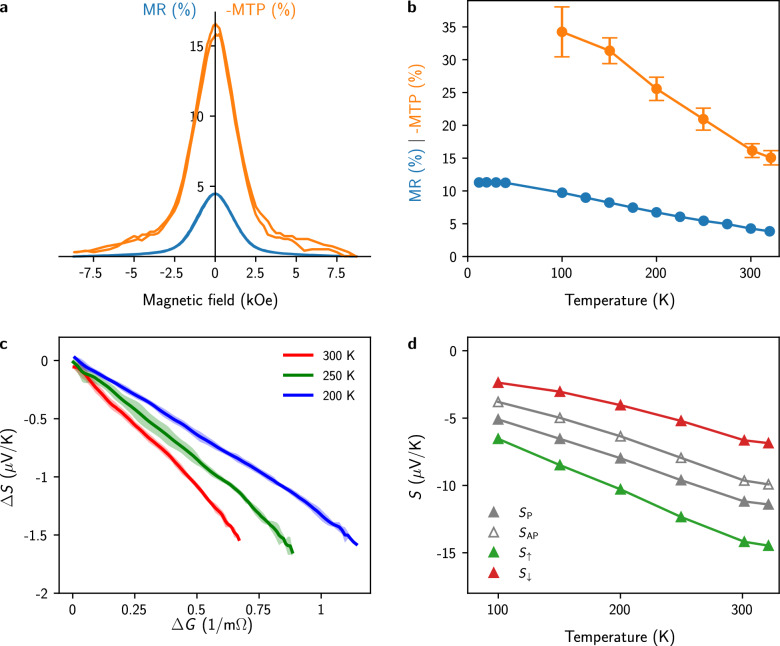
Giant magneto-thermopower in Fe/Cu nanowire networks. (**a**) Room-temperature magnetoresistance (left side, in blue) and magneto-Seebeck (right side, in orange) curves obtained by sweeping an external magnetic field along the in-plane direction of a Fe(7 nm)/Cu(10 nm) multilayer NW network. (**b**) Variation with temperature of the MR and MTP ratios. (**c**) Linear variation of $\Delta S(H) = S(H) - S_\text {AP}$ vs. $\Delta G = 1/R(H) - 1/R_\text {AP}$ at different measured temperatures, illustrating the Gorter–Nordheim characteristics for the interconnected Fe/Cu NW sample (Eq. 6). The shaded area shows the data uncertainty. (**d)** Temperature variation of the measured Seebeck coefficients at zero applied fields $S_\text {AP}$ and at saturating magnetic fields $S_\text {P}$ along with the corresponding calculated spin-dependent Seebeck coefficients $S_\uparrow$ and $S_\downarrow$ using Eqs. (9) and (10). The error bars reflect the uncertainty of the electrical and temperature measurements and is set to two times the standard deviation, gathering 95$\%$ of the data variation.

Figure 5a shows the RT magnetoresistance (MR) and magneto-thermoelectric (MTP) measurements for Fe(7 nm)/ Cu(10 nm) ML NW network. Here, MR(*H*) $=$ ($R(H) - R_\text {sat})/R_{0}$, with *R*(*H*) being the resistance at a given external magnetic field value *H*, $R_\text {sat}$ the resistance at the saturation field, and $R_{0}$ the resistance at $H =$ 0. Similarly, MTP(*H*) $=$ ($S(H) - S_\text {sat})/S_{0}$, with *S*(*H*) the Seebeck coefficient at a given external magnetic field value *H*, $S_\text {sat}$ the Seebeck coefficient at saturation field, and $S_{0}$ the Seebeck coefficient at $H =$ 0. Due to the negative values of the Seebeck coefficient in Fe/Cu multilayers, the MTP values are also negative. As shown in Fig. 5a, the MTP effect ($\sim$ 16$\%$) at RT is about 4 times larger than the corresponding MR effect ($\sim$ 4$\%$). This is in contrast to the measurements previously performed on the 3D NiCo/Cu and Co/Cu NW networks, where the amplitudes of the MR and MTP effects are similar at RT^25,26^. However, a much larger MTP effect relative to the MR effect was found in NiFe/Cu NW networks with low concentration of Fe in the alloy^45^. Figure 5b shows the temperature dependence of the MR and MTP ratios for the interconnected Fe/Cu NWs. As seen, the MR ratio shows a monotonic increase before reaching a plateau at MR $\sim$ 11$\%$ at low temperatures. This is expected because of the saturation of the resistivity at low temperatures and the vanishing of the spin mixing effect. On the other hand, the value of −MTP shows a pronounced increase with decreasing temperature, with values exceeding 30$\%$ around $T =$ 100 K. This behavior is also consistent with previous temperature dependency measurements of the MTP for NiFe/Cu ML NW networks^27^.

The fact that the thermopower is dominated by electron diffusion in Fe/Cu ML NW networks is also supported by the linear variation between the field-dependent Seebeck coefficient *S*(*H*) and the inverse of the field-dependent resistance 1/*R*(*H*), as shown in Fig. 5c at some selected temperatures. These curves correspond to Gorter–Nordheim plots for the diffusion thermopower in metals and alloys^1^. In the case of magnetic multilayers, the Gorter–Nordheim relation can be written as^25,46^:

6 $$\begin{aligned} S(H) = A + \frac{B}{R(H)}\text {,} \end{aligned}$$

where *A* $=$ $(S_{0}R_{0} - S_\text {sat}R_\text {sat})/(R_{0} - R_\text {sat})$ and *B* $=$ $R_{0}R_\text {sat} (S_\text {sat} - S_{0})/(R_{0} - R_\text {sat})$. Similar characteristics have previously been reported for interconnected Co/Cu, Co$_{50}$Ni$_{50}$/Cu and NiFe/Cu NW networks^25–27^.

Using the simple two-current series-resistor model for perpendicular transport of electrons through magnetic multilayers and assuming that the layers of the multilayer stack are thin compared to the spin-diffusion lengths (long SDL limit), the corresponding thermopowers $S_\text {AP}$ and $S_\text {P}$ for antiparallel (AP) and parallel (P) configurations are given by^46,47^

7 $$\begin{aligned} S_\text {AP} = \frac{S_\uparrow \rho _\uparrow + S_\downarrow \rho _\downarrow }{\rho _\uparrow + \rho _\downarrow } \end{aligned}$$

and

8 $$\begin{aligned} S_\text {P} = \frac{S_\uparrow \rho _\downarrow + S_\downarrow \rho _\uparrow }{\rho _\uparrow + \rho _\downarrow }\text {,} \end{aligned}$$

where $\rho _\uparrow$, $\rho _\downarrow$ and $S_\uparrow$ , $S_\downarrow$ are separate resistivities and Seebeck coefficients for majority and minority spin channels. Therefore, using Eqs. (7) and (8), the Seebeck coefficients for spin-up and spin-down electrons can be written as^25^

9 $$\begin{aligned} S_{\uparrow } = \frac{1}{2} \big [S_{\text {AP}}\big (1-\beta ^{-1}\big ) + S_{\text {P}}\big (1+\beta ^{-1}\big ) \big ] \end{aligned}$$

and

10 $$\begin{aligned} S_{\downarrow } = \frac{1}{2} \big [S_{\text {AP}}\big (1+\text {MR}^{-1/2}\big ) + S_{\text {P}}\big (1-\text {MR}^{-1/2}\big ) \big ]\text {,} \end{aligned}$$

where $\beta = (\rho _\downarrow - \rho _\uparrow )/(\rho _\downarrow + \rho _\uparrow )$ denotes the spin asymmetry coefficient for resistivity. CPP-giant magnetoresistance (GMR) effects are observed in the Fe/Cu NWs, as illustrated in Fig. 5a. For a CPP-GMR system with individual layer thicknesses close to 10 nm, it was found that the contribution of bulk scattering is significantly larger than the contribution of interface scattering^16^ so that MR $\sim \beta ^2$ where MR $= (R_\text {AP}-R_\text {P})/R_\text {AP}$ with $R_\text {AP}$ and $R_\text {P}$ the electrical resistance for the antiparallel (or random in long SDL limit^48^) and parallel arrangements, respectively. Figure 5d shows the temperature evolutions of $S_\text {AP}$, $S_\text {P}$, $S_\uparrow$ and $S_\downarrow$ for interconnected Fe/Cu NWs. At room temperature, the estimated values are $\beta \approx$ 0.21, $S_\uparrow = -$ 14.2 $\upmu$V/K and $S_\downarrow = -$6.6 $\upmu$V/K. Below RT, the various Seebeck coefficients decrease almost linearly with decreasing temperature, which is also indicative that diffusion thermopower is the dominant mechanism. Similar results were obtained on Co/Cu, CoNi/Cu and NiFe/Cu ML NW networks^25,27,49^. From the analysis, the estimated RT value for ($S_\uparrow - S_\downarrow$) of − 7.6 $\upmu$V/K for Fe/Cu NWs is similar to that previously reported for Co/Cu^26^ and CoNi/Cu^25^ NWs although it is much smaller than for NiFe/Cu multilayer NWs with low amount of Fe content in the alloy ($S_\uparrow - S_\downarrow$ $\sim$ − 20 $\upmu$V/K at RT for Ni$_{97}$Fe$_{3}$/Cu NWs, see^45^).

## Conclusion

In this work, we determined the respective magnon-drag and thermal diffusion contributions to the thermopower of pure Fe nanowires and nanowires based on dilute FeCu and FeCr alloys having Cu and Cr concentrations in the range of 1 to 10 atomic percent. The interconnected ferromagnetic nanowire networks were grown by electrodeposition within 3D porous polymer membranes. Temperature dependent measurements were also performed on a Fe(7 nm)/Cu(10 nm) multilayer nanowire sample exhibiting giant magnetoresistance and giant magneto-Seebeck effects. The Fe/Cu multilayer was obtained by a pulsed electrodeposition process from a single electrolyte solution. All measurements were performed in the direction of the film plane, while limiting electrical and thermal currents along the NW segments of the interconnected NW network. The thermopower of 45 nm diameter Fe NWs shows the same positive values and temperature dependence as bulk iron with a maximum in the *S*(*T*) curve around $T =$ 200 K and a value of 14 $\upmu$V/K at room temperature. The introduction of copper impurities leads to a sharp decrease in the total measured thermopower which becomes negative for the Fe$_{90}$Cu$_{10}$ alloy. The results obtained on FeCu NWs are similar to those previously obtained on dilute FeCo and FePt bulk alloys. The linear dependencies of the thermopower observed in the high temperature range show very similar negative slopes for pure iron and the different alloys and allow an estimation of the diffusion thermopower. For pure iron, the estimated diffusion thermopower value of − 15 $\upmu$V/K at $T =$ 300 K is significantly greater than that estimated in Blatt’s pioneering work ($\sim$ $-5$ $\upmu$V/K). This value is however lower than those measured on pure Ni and Co, respectively near − 20 $\upmu$V/K and − 30 $\upmu$V/K at $T =$ 300 K. From our analysis, it follows that the contribution of the magnon-drag effect is dominant in Fe, reaching maximum positive values close to 30 $\upmu$V/K at room temperature. Our estimate of the magnon-drag contribution to the *S*(*T*) curve between 70 and 320 K in pure Fe NWs agrees very well with previous estimates at very low temperatures in bulk Fe. Our results clearly demonstrate the reduction of magnon-drag thermopower in nanowire networks made of dilute FeCu and FeCr alloys with increasing impurity concentration. For both types of nanowire alloys, the room-temperature magnon-drag thermopower drops to about 10 $\upmu$V/K at 10$\%$ impurity content. It also appears that the introduction of Cr impurities into Fe nanowires not only reduces the magnon-drag thermopower but also strongly affects the contribution from charge carrier diffusion. Although in dilute NiCr alloys, sign inversion of the diffusion thermopower from negative to positive was even observed, the impurity effect in FeCr NWs leads to a pronounced drop of the negative diffusion thermopower, as also recently found in CoCr CNWs. The thermopower of the Fe(7 nm)/Cu(10 nm) multilayer nanowires is negative with linear temperature dependence and obeys the Gorter–Nordheim rule, indicating that the contribution from charge carrier diffusion is the dominant mechanism contributing to the thermopower in magnetic multilayers. A negative thermopower of about − 11 $\upmu$V/K is measured at room temperature, which can be related remarkably well to a diffusive thermopower of about − 15 $\upmu$V/K for the constituent layers of Fe. Giant magnetoresistance and magneto-Seebeck effects were measured with amplitudes of 4$\%$ and 16$\%$ at room temperature, respectively. The MR and MTP ratios reach about 10$\%$ and 35$\%$ at $T =$ 100 K, respectively. For Fe/Cu NWs, the estimated RT value of the spin-dependent Seebeck coefficient ($S_\uparrow - S_\downarrow$) of − 7.6 $\upmu$V/K is similar to that previously reported for Co/Cu and CoNi/Cu NWs.

## Data Availability

All data generated during and analysed during the current study are available from the corresponding author on reasonable request.
